# Effects of simulation with problem-based learning (S-PBL) on nursing students’ clinical reasoning ability: based on Tanner’s clinical judgment model

**DOI:** 10.1186/s12909-023-04567-9

**Published:** 2023-08-24

**Authors:** Hae Kyoung Son

**Affiliations:** Department of Nursing, Eulji University, Seongnam city, 13135 Republic of Korea

**Keywords:** Clinical reasoning, Nursing education, Nursing students, Problem-based learning, Simulation

## Abstract

**Background:**

Clinical reasoning ability, a complex cognitive and metacognitive process, is a crucial core competency required in nursing practice. Therefore, undergraduate nursing students should be provided with nursing education to strengthen their clinical reasoning ability based on real-life nursing scenarios.

**Methods:**

This study was conducted using a quasi-experimental single-group pretest–posttest design. Three sessions (lasting three hours each) of Simulation with Problem-Based Learning (S-PBL) using high-risk obstetrics-gynecology scenarios were provided to 71 third-year nursing students of a university. The sessions were conducted from September to December 2022, and they aimed to strengthen their clinical reasoning ability. For data collection, an online survey was conducted using Rubric for Clinical Reasoning and learning satisfaction evaluation tool. Data were analyzed using descriptive statistics and repeated measures analysis of variance in SPSS.

**Results:**

The mean score of clinical reasoning ability significantly increased from 29.42 (standard deviation: 4.62) out of 40 points in the pre-test to 32.28 (4.36), 33.44 (5.35), and 33.80 (5.91) after the first, second, and third S-PBL sessions, respectively (F = 61.668, p < .001). The learning satisfaction score was as high as 107.04 (12.66) out of 120 points.

**Conclusion:**

This S-PBL program is an effective nursing education strategy to strengthen nursing students’ clinical reasoning ability. Future studies must examine learner variables and standardize the S-PBL design and operation process by comparison to a traditional teaching approach and a higher range of clincal reasoning ability.

**Supplementary Information:**

The online version contains supplementary material available at 10.1186/s12909-023-04567-9.

## Introduction

With the increasing demand for high-risk obstetrics-gynecology (Ob/Gyn) nursing, nurses’ clinical reasoning ability must derive optimal health outcomes for patients [1, 2]. Considering this need, the Korean Accreditation Board of Nursing Education defined clinical reasoning ability as a core competency for nursing graduates as academic outcomes to emphasize quality management, improve nursing education programs based on its performance-oriented education system, and produce students with competencies required in clinical practice [3].

Clinical reasoning refers to a decision-making process to interpret or draw conclusions regarding patients’ needs, interests, and health problems and determine alternative actions by collecting and analyzing related information— a complex cognitive and metacognitive process [4–6]. Specifically, it is a cognitive process and strategy used by nurses to identify critical patient data, diagnose their actual and potential problems, and make clinical decisions to solve problems resulting in positive outcomes [7–13].

Generally, clinical reasoning and clinical judgment are used interchangeably as well as for problem-solving, decision-making, and critical thinking [14]. While clinical judgment focuses on the results of a nurse’s interpretation or judgment of a patient’s condition, clinical reasoning is primarily concerned with the process leading to that judgment. Clinical reasoning includes a characteristic pattern of the reflection process to gauge understanding of a given clinical situation, identify problems and needs, prepare alternative courses of action, and choose the appropriate alternative considering the evidence [4]. Therefore, this study approached nursing students’ clinical reasoning ability with an emphasis on strengthening their clinical reasoning. This differs from previous studies, wherein clinical reasoning ability was treated as an outcome variable [14].

Tanner [4] noted that clinical reasoning ability is a core competency essential for all nurses, proving they are professional nurses. However, despite the need to equip themselves with professional nursing competencies during their undergraduate years, nursing students have limited opportunities to obtain direct nursing experiences in high-risk Ob/Gyn cases using their clinical reasoning ability [15–18]. Nursing educators must guide nursing students to acquire and strengthen their clinical reasoning ability by providing experiential learning to train clinical reasoning based on diverse and unique clinical nursing situations to equip them with nurse competencies [4, 10, 12, 13, 15–18].

In this context, this study was conducted to apply the Simulation with Problem-Based Learning (S-PBL) program based on the Clinical Judgment Model proposed by Tanner [4] to strengthen nursing students’ clinical reasoning ability as a conceptual framework for teaching and evaluate this as regular undergraduate nursing curriculum. This program, S-PBL, is a learning method combining simulation training and PBL, wherein a team of students experiences the learner-centered process of jointly solving a multidisciplinary problem [19–23]. As an alternative to nursing education, S-PBL is a highly effective intervention for improving nursing competencies, such as clinical reasoning, by integrating theoretical learning into clinical practice through repetitive experiential learning, immediate feedback, evaluation, and reflection based on extensive real-life nursing scenarios [24–28]. Specifically, it efficiently improves their clinical reasoning ability, allowing them to reflect on the problem-solving process or integrate clinical judgment into debriefing [4, 28]. Therefore, S-PBL’s educational effect on strengthening nursing students’ clinical reasoning ability was examined by providing them with S-PBL sessions using high-risk Ob/Gyn nursing scenarios during nursing education and repeatedly measuring their clinical reasoning ability.

This study aimed to examine the effects of S-PBL on nursing students’ clinical reasoning ability by comparing and analyzing how it is strengthened based on repeatedly measured data and to identify their learning satisfaction. The following hypotheses were examined:

### Hypothesis 1-1

Nursing students’ clinical reasoning ability will be higher after participation in S-PBL than before.

### Hypothesis 1-2

Nursing students’ clinical reasoning ability will gradually increase with repeated participation in S-PBL.

### Hypothesis 2

Nursing students’ learning satisfaction will be high after participation in S-PBL.

## Materials and methods

### Study design and participants

**Fig. 1:**
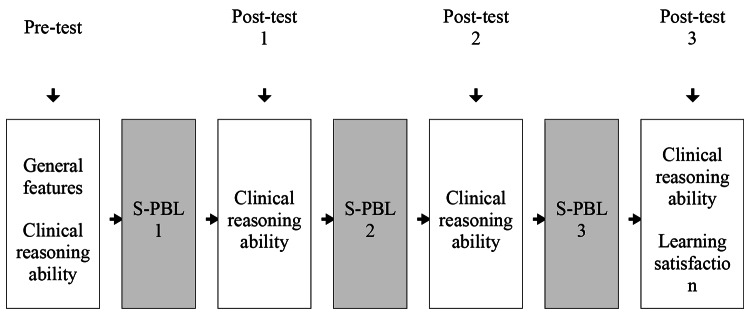
Research design

This study was conducted using a quasi-experimental single-group pretest–posttest design with repeated measures analysis of variance (ANOVA) (Fig. 1).

The participants were third-year nursing students of a university located in S city (a metropolitan area) taking the Maternity Nursing II course using S-PBL to strengthen clinical reasoning ability based explicitly on high-risk Ob/Gyn nursing scenarios. The participants were selected through convenience sampling from 89 nursing students participating the Maternity Nursing II course during the semester. Nursing students who understood the purpose of this study during the subject orientation and voluntarily agreed to participate were selected. Selection criteria were the completion of the mandatory Maternity Nursing I course as a prerequisite course, prior knowledge of normal pregnancy, childbirth, and postpartum care, and voluntary consent to participate in this study.

**Fig. 2:**
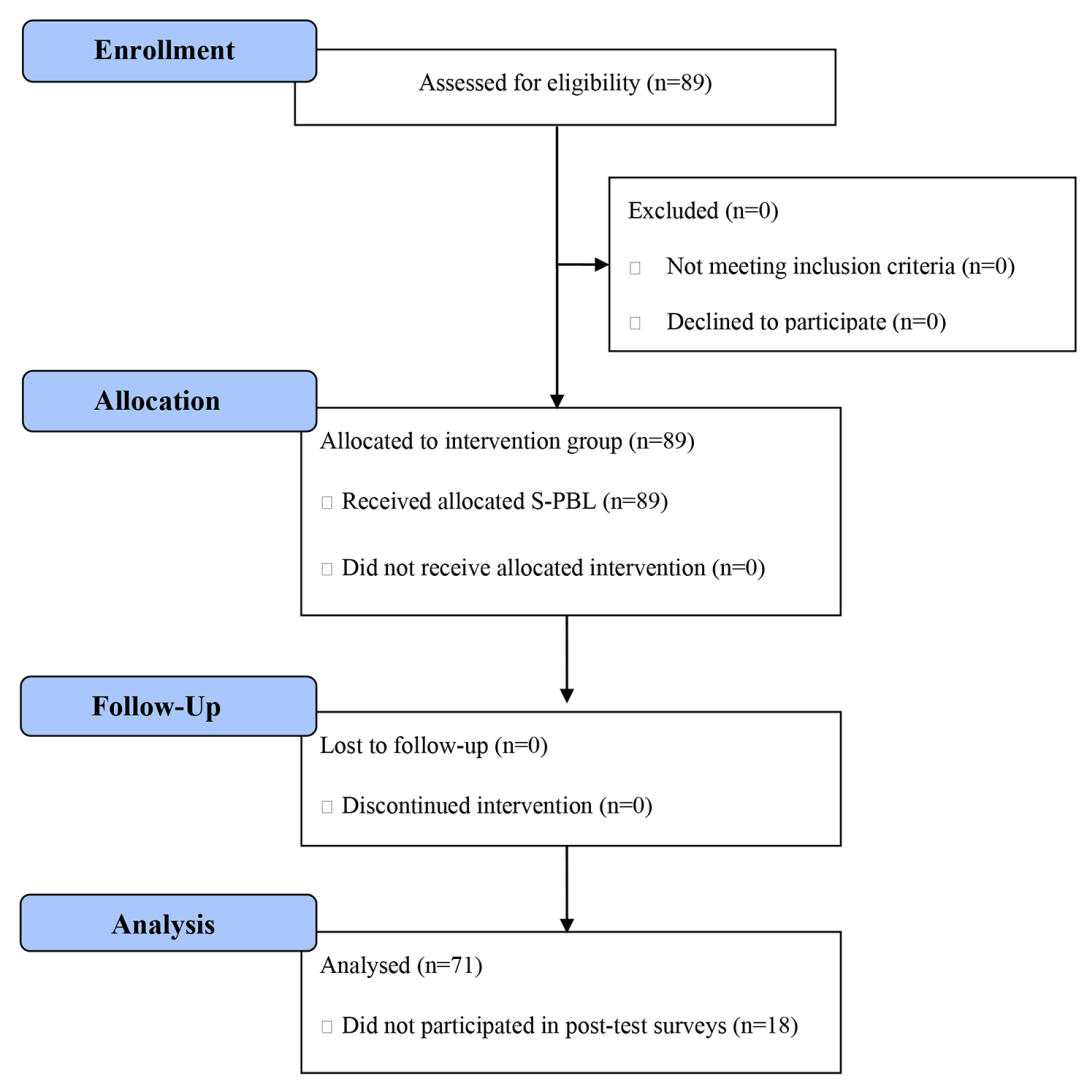
Flow diagram

In a study using repeated measures ANOVA, a sample size of 15 to 30 was reported to be appropriate [23]. The minimum required sample size of 36 was calculated for 1 × 4 repeated measures ANOVA using the G*Power software program (Version 3.1.9.4, Franz Faul, Universität Kiel, Germany) (RRID:SCR_013726; http://www.gpower.hhu.de (6 February 2019)) at significant level α = 0.05, power = 0.95, and effective size = 0.25 [29, 30]. However, considering this study is part of the curriculum, all 89 nursing students taking the Maternity Nursing II course were enrolled without considering a dropout rate. After excluding 18 students who dropped out from failure to complete the questionnaire for one or more post-test surveys, data from 71 students were included in final analysis, which met the required minimum sample size (Fig. 2).

### Research tools

#### General characteristics

As variables of general characteristics, age, gender, religion, perceived general health status, reason for majoring in nursing, Grade Point Average (GPA), residential status, and satisfaction with majoring in nursing were examined. These variables were considered influencing factors of academic burnout of nursing students and learning transfer such as clinical judgment in nursing education [31, 32]. Accordingly, these were examined as variables of general characteristics.

#### Clinical reasoning ability

Nursing students’ clinical reasoning ability was measured using Rubric for Clinical Reasoning [14]. This Korean tool is a criteria scale for evaluating students’ performance using Rubric’s key concepts, developed based on the four aspects of the Clinical Judgment Model proposed by Tanner [4], including noticing (understanding patients’ condition & related data), interpreting (interpreting patients’ condition & related data), responding (dealing with patients), and reflecting (reflection on nursing action) [2, 14]. The Rubric for Clinical Reasoning comprises ten items (evaluation elements): Noticing (three evaluation elements, including “patient observation,” “noticing any change in patients’ condition,” and “search for patient information”); Interpreting (two evaluation elements, including “prioritization of nursing care” based on clinical reasoning and “organized interpretation of related data”); Responding (three evaluation elements, including nurse’s “attitude” toward the patient, “communication,” and “nursing intervention”); Reflecting (two evaluation elements, including “reflection on decision-making for nursing action” while providing care and “reflection on nursing action itself” after providing care). Each element for the qualitative performance was self-rated on a four-point Likert scale (1 = beginning, 2 = developing, 3 = achieving, and 4 = exemplary), with the total scores ranging from 10 to 40 points, wherein a higher total score indicated a higher level of clinical reasoning ability. Clinical reasoning ability was categorized into four levels following the Rubric scores proposed by Kim and Ko [14]: beginning (10 to < 14 points), developing (15 to < 25 points), achieving (25 to < 35 points), and exemplary (35–40 points). Cronbach’s ⍺ of this tool was .97 in the study by Kim and Ko [14] and .95 in this study, ranging from .80 to 0.89 for each aspect of Rubric for Clinical Reasoning: noticing (Cronbach’s ⍺ .89), interpreting (Cronbach’s ⍺ .80), responding (Cronbach’s ⍺ .89), and reflecting (Cronbach’s ⍺ .81).

#### Learning satisfaction

Learning satisfaction was measured using the learning satisfaction evaluation tool for nursing students developed by Yoo and Yoo [23]. This Korean tool comprises 24 items for rating the level of satisfaction with nursing students’ perceived attitude toward lectures, degree of lecture preparation by professors, delivery and content of lectures, and evaluation of academic achievements. Each item is rated on a five-point Likert scale (1 = not satisfied, 5 = very satisfied), with the total score ranging from 24 to 120 points, wherein a higher total score indicates a higher level of learning satisfaction. Cronbach’s ⍺ of this tool was .94 in the study by Yoo and Yoo [23] and .95 in this study.

### S-PBL

The intervention, S-PBL, was developed as a nursing education program to strengthen nursing students’ clinical reasoning ability using high-risk Ob/Gyn nursing scenarios in the Maternal Nursing II course. The nursing process of the S-PBL program is based on four aspects (i.e., noticing, interpreting, responding, and reflecting) of the clinical judgment process proposed by Tanner [4] in individual or small-group activities (Table 1). Each stage is associated with a cognitive process performed by the students. Noticing involves observing patients, monitoring changes in their conditions, exploring subjective and objective patient information, and effectively recognizing the given nursing situation using task performance plans based on the knowledge and skills acquired through nursing education. Interpreting involves setting patient care priorities by collecting sufficient data to understand the nursing situation and conducting an integrated interpretation of patient-related data based on persona modeling. Responding involves applying the nursing process based on appropriate nursing care decisions. Reflecting involves writing individual reflection journals based on a self-reflection of whether all aspects of the nursing process were appropriate at each of the previous clinical judgment stages and sharing them with other students.

**Table 1:** The contents of S-PBL

Session	Stage	Contents	Duration (min)
S-PBL 1	Topics Education Noticing Interpreting Responding Reflecting	Premature rupture of membranes & Preterm labor Orientation & High-risk Ob/Gyn nursing lectures Overview of cases & Task performance plans Persona modeling activity & Group discussion Nursing process & Peer role-play Reflection journals & Sharing the experience & Post-test 1	Total 180 30 30 30 60 30
*(Intervals : two weeks)*			
S-PBL 2	Topics Education Noticing Interpreting Responding Reflecting	Postpartum hemorrhage Orientation & High-risk Ob/Gyn nursing lectures Overview of cases & Task performance plans Persona modeling activity & Group discussion Nursing process & Peer role-play Reflection journals & Sharing the experience & Post-test 2	Total 180 30 30 30 60 30
*(Intervals : two weeks)*			
S-PBL 3	Topics Education Noticing Interpreting Responding Reflecting	Uterine fibroids Orientation & High-risk Ob/Gyn nursing lectures Overview of cases & Task performance plans Persona modeling activity & Group discussion Nursing process & Simulation (Papanicolaou smear test) Reflection journals & Sharing the experience & Post-test 3	Total 180 30 30 30 60 30

The students attended three S-PBL sessions (three hours each) in individual and small-group activities as a part of the lectures covering the related topics in the 15th week (30 h total) of the Maternity Nursing II course. Each S-PBL session was provided at regular intervals (at two weeks intervals) except for the exams and the instructor provided new exposure of the content based on the high-risk Ob/Gyn nursing knowledge every session to minimize the history, maturation, and repeated testing effect [33]. Individual activities involved acquiring nursing knowledge through lectures, writing task performance plans to apply the knowledge acquired based on the topic-related high-risk Ob/Gyn nursing scenarios presented by the instructor, including premature rupture of membranes, premature labor, postpartum hemorrhage, and uterine fibroids, as pre-session activities, and presenting self-reflection and self-evaluation through reflection journals as wrap-up activities. The students were instructed to spend 30 min writing an individual task performance plan on how to approach the given nursing task in the order of (i) organizing the already known facts in a given problematic situation, (ii) formulating hypotheses, solutions, or nursing goals in the problem-solving/planning stage, (iii) exploring additional information required to solve the problem, and (iv) setting up nursing intervention plans seeking problem-solving ideas. Subsequently, they were instructed to share their individually prepared approaches to problem-solving in small groups of four to five students for approximately 30 min.

In the subsequent small-group activity (duration: 1 to 1.5 h), the instructor provided cues necessary for the small-group activity in the form of photos, video clips, and voice recordings containing detailed nursing-related information based on the prioritized nursing interventions selected from individual task performance plans in the small-group presentations and evaluations. Data such as photos and videos for S-PBL were prepared beforehand in the nursing college’s simulation room using high-fidelity patient simulators (Gaumard® Noelle® S554.100, Miami, FL, USA) based on the scenarios developed by the researcher. In the first and second S-PBL sessions, students performed peer role-play based on the nursing information provided. In the third S-PBL session, they were allowed to solve nursing problems in small groups by directly performing the Papanicolaou smear test on female genital mannequins designed for cervical examination.

The high-risk Ob/Gyn nursing scenarios for S-PBL were based on the Korean Society of Obstetrics and Gynecology, women’s health nursing textbooks and literature, and Ob/Gyn nursing applied in outpatient settings. The scenarios were constructed to reproduce high-risk Ob/Gyn nursing situations, such as premature rupture of memebranes, premature labor, postpartum hemorrhage, and uterine fibroids, and allow communication with healthcare workers, including nurses and patients or caregivers and nursing education. The S-PBL sessions were structured to allow nursing students to communicate with one another through learner-directed small-group activities, separate from knowledge-transfer lectures, and to modify the current or begin a new learning process gradually, leading from basic to intensified to integrated levels. The instructor facilitated students to participate in individual and group activities actively, led the S-PBL session, and mediated when there were achievement gaps at the individual student or group level. In the final stage of each S-PBL session, students were instructed to write individual reflection journals and share their impressions between participants and learning experiences through presentations for approximately 30 min, followed by the instructor’s wrap-up feedback and outcome evaluation.

### Data collection and analysis

Data were collected from September to December 2022 from nursing students taking the Maternity Nursing II course at a university in S city (a metropolitan area). Before data collection, the researcher explained (verbally) the purpose and study procedure and the voluntary participation. Data were collected at the baseline (pretest) and immediately after each S-PBL session (three repeated posttests) using a questionnaire requiring five minutes. Data was collected online via Google survey forms in a structured self-report questionnaire. The survey was completed anonymously and individually by participants in a unsupervised manner to minimize any potential coercion, pressure, and bias within the data collection process. They were given a small token of appreciation for participation (stationery worth 1,000 won).

Data analysis was performed using the IBM SPSS Statistics ver. 22.0 program (IBM Co., Armonk, NY, USA). The participants’ general characteristics were analyzed via descriptive statistics, the reliability of the measurement tool via Cronbach’s ⍺ coefficient, and the intervention effect via repeated measures ANOVA.

### Ethical considerations

This study was conducted according to the guidelines of the Declaration of Helsinki and approved by the institutional review board of Eulji University (IRB No. EU22-50). The participants could verbalize their intention to decline participation at any time, which was explained in detail before the study’s commencement. To protect participants’ privacy, an online platform was used for the survey.

## Results

### General characteristics

Table 2 outlines the participants’ general characteristics: Mean age was 23.48; female students far outnumbered male students; most had no religion, followed by Protestants and Catholics; most perceived their health as “good;” the reason for studying nursing included high employment rates, aptitude, other’s recommendation, and good image of a nurse, in that order; most had above-average GPA in the previous semester; most were living in their parent’s homes; most had a moderate or high level of satisfaction with majoring in nursing. There were no confounding effects of the general characteristics on clinical reasoning ability as a main outcome (p > .05).

**Table 2:** General characteristics (N = 71)

Characteristics	Categories	Mean (SD) / n (%)	t (p)
Age (year)		23.48 (2.51)	0.833 (0.408)
Gender	Female	53 (74.6)	0.798 (0.428)
	Male	18 (25.4)	
Religion	Protestant	16 (22.5)	1.322 (0.191)
	Buddhist	3 (4.2)	
	Catholic	10 (14.1)	
	None	42 (59.1)	
Perceived general health status	Good	56 (78.9)	0.077 (0.939)
	Moderate	14 (19.7)	
	Poor	1 (1.4)	
Reason for majoring in nursing	High employment rates	19 (26.8)	0.793 (0.431)
	Aptitude	18 (25.4)	
	Considering high school GPA	3 (4.2)	
	Other’s recommendation	14 (19.7)	
	Good impression of a nurse	14 (19.7)	
	Others	3 (4.2)	
GPA	≧ 4.0 and < 4.5	23 (32.4)	0.199 (0.843)
	≧ 3.5 and < 4.0	22 (31.0)	
	≧ 3.0 and < 3.5	20 (28.2)	
	< 3.0	6 (8.5)	
Residential status	With family	51 (71.8)	1.438 (0.155)
	With friends	11 (15.5)	
	Alone	7 (9.9)	
	Other	2 (2.8)	
Satisfaction with majoring in nursing	Satisfying	27 (38.0)	0.089 (0.929)
	Moderate	36 (50.7)	
	Not satisfying	8 (11.3)	

### Intervention effects

#### Hypothesis 1-1

**Nursing students’ clinical reasoning ability will be higher after participation in S-PBL than before.**

#### Hypothesis 1-2

**Nursing students’ clinical reasoning ability will gradually increase with repeated participation in S-PBL.**

**Fig. 3:**
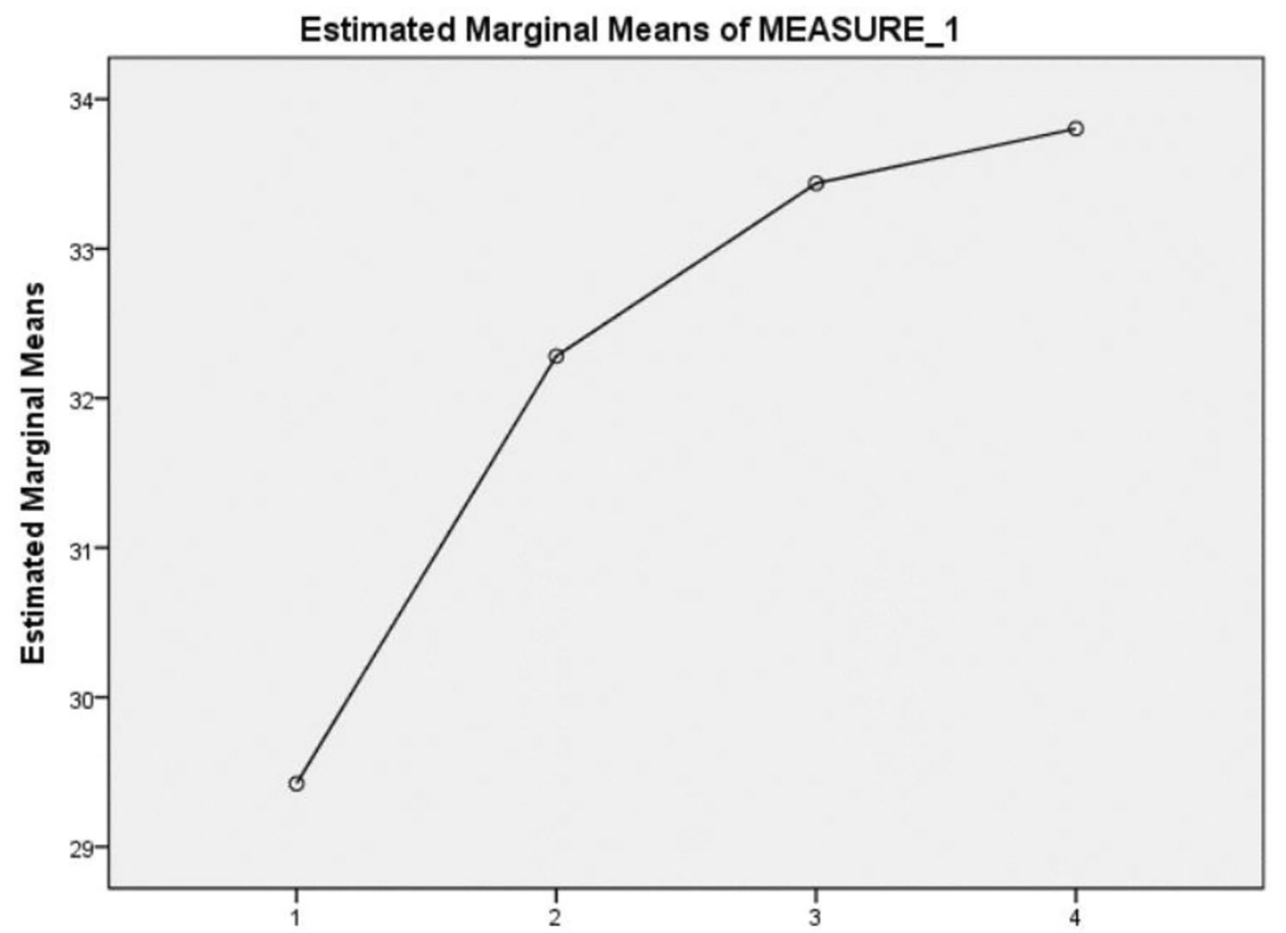
Profile plots of clinical reasoning ability *Note*. 1 = Pre-test; 2 = Post-test 1; 3 = Post-test 2; 4 = Post-test 3.

The mean pre-test clinical reasoning ability score was 29.42 (standard deviation: 4.62), which gradually increased to 32.28 (4.36) after the first S-PBL session, 33.44 (5.35) after the second session, and 33.80 (5.91) after the third session. The repeated measures ANOVA confirmed the sphericity assumption in Mauchly’s test of sphericity (Huynh-Feldt = 0.930) and significant clinical reasoning score differences as the sessions progressed from the pre-test to the S-PBL post-test sessions one through three (F = 61.668, p < .001), with the post-test scores significantly higher than the pre-test score and the scores increasing after each S-PBL session (Table 3; Fig. 3), thus supporting Hypothesis 1–1 and Hypothesis 1–2, respectively.

#### Hypothesis 2

**Nursing students’ learning satisfaction will be high after participation in S-PBL**.

The participants’ learning satisfaction after the S-PBL sessions was as high as 107.04 (standard deviation: 12.66) out of 120 points, supporting Hypothesis 2.

**Table 3:** Descriptive statistics of clinical reasoning ability (N = 71)

Variable (range)	Mean (SD)				F (p)	Pairwise comparisons
	Pre-test^a^	Post-test 1^b^	Post-test 2^c^	Post-test 3^d^		
Clinical inference (10–40)	29.42 (4.62)	32.28 (4.36)	33.44 (5.35)	33.80 (5.91)	61.668 (0.000)	a < b < c,d

*Note.* a, b, c, d; Pretest and Post-test 1, 2, 3 for Pairwise comparisons, respectively, SD; Standard Deviation

## Discussion

This study provided essential data for setting up effective nursing education strategies by applying S-PBL to strengthen nursing students’ clinical reasoning ability based on the Clinical Judgment Model and verifies its educational effect through repeated measures. The course of S-PBL was designed following a combination of simulation-based learning approaches and problem-based learning. In each S-PBL session the nursing students challenged to learn and solve different types of the high-risk Ob/Gyn nursing problems. The nursing students worked individually, then in groups of four to five students and then sharing their findings of learning transfer. The three S-PBL sessions in individual and small-group activities were similar to a three-phased problem-based learning approach that combined simulation-based learning activities [34, 35]. In this study, S-PBL improved nursing students’ clinical reasoning ability, and they showed a high level of learning satisfaction after S-PBL participation.

Compared to the pre-test at baseline (before participating in S-PBL), nursing students’ clinical reasoning ability significantly improved after participation, with the mean scores gradually increasing as the S-PBL session progressed. To understand the learner’s competency improvement process for learning transfer, its effect should focus on the transfer process wherein the learner acquires knowledge and applies it [36]. Hence, this study is significant because it repeatedly applied the educational program to nursing students and evaluated their clinical reasoning ability as the learning transfer stage progressed. Notably, the mean clinical reasoning score of senior nursing students of a four-year university was 14.26 (5.44) points (full score: 40 points) in a previous study [14], which is the beginning level (10 to less than 15 points). In the study by Lasater [2], using the Lasater clinical judgment rubric (LCJR), similar to the Rubric for Clinical Reasoning used in this study, the mean score of junior nursing students converted to a 40-point scale was 20.89 (5.52) points, which corresponds to the developing level (15 to < 25 points). The mean score of senior nursing students measured using the Korean version of the LCJR in a study by Shin, Park, and Shim [37] was 27.02 (5.35) points (full score: 40 points), corresponding to the achieving level (25 to < 35 points). The four phases of Tanner’s [4] Clinical Judgment Model, including noticing, interpreting, responding, and reflecting, also helped formulate the LCJR [2]. Comparing the scores of the clinical reasoning ability of the junior nursing students who participated in this study with those of the above-mentioned studies, all post-test scores were in a higher range of the achieving level (25 to < 35 points) with 32.28 (4.36), 33.44 (5.35), and 33.80 (5.91) after the first, second, and third S-PBL sessions, respectively, thus demonstrating the effectiveness of S-PBL as a nursing education strategy. This finding was interpreted as a result of motivating the students, promoting knowledge sharing and fostering system thinking by using various forms of photos, video clips, and voice recordings containing detailed nursing-related information, similar to that of a previous study by analyzing qualitative data using questionnaries and ain-deep interviews after problem-based learning approach that combined simulation-based learning activities [35].

However, caution is warranted in interpreting this study’s results, given that no direct comparison with the control group could be made to consider the influencing factors of clinical reasoning ability. Using a study design involving repeated measures ANOVA performed on the same participants in a time series after each intervention session, the same group acted as the control group. Repeated measures ANOVA is a powerful analysis method that supports research results with a relatively small sample size by increasing the statistical power of analysis and reducing the error variance [38]. Hence, its use may have increased the validity of this study’s results as a quasi-experimental single-group study of junior nursing students of a four-year university conducted as part of the nursing curriculum. Additionally, it is necessary to investigate on the long-term impacts of problem-based learning in nursing education according to a systematic review and meta-analysis [39]. Since this study examined only the short-term effect of S-PBL, it is necessary to evaluate how the education effect is maintained or how clinical reasoning is applied to actual nursing practice, which is the final stage of learning transfer.

Furthermore, learning satisfaction after S-PBL was as high as 107.04 (12.66) out of 120 points (8.92 when converted to a 10-point scale), which is higher than Yoo and Yoo [23]’s mean learning satisfaction of 98.23 (10.56) in their study. This may be because the constructivist learning approach emphasizes authenticity and contextualization [40]. That is, S-PBL enhances learning satisfaction by positively influencing learners’ interest in and attention to the learning contents and class participation because it facilitates understanding and problem-solving related to the acquired knowledge by constructing and reconstructing knowledge through continuous interactions with the given reflective environment and nursing situations similar to actual clinical settings [23, 40].

This study has some limitations. Since this is a quasi-experimental study, with its participants as nursing students enrolled in a specific university course in nursing curriculum recruited by convenience sampling, caution is warranted in interpreting its results, and repeated studies must test its generalizability by recruiting nursing students with different characteristics including a higher range of clinical reasoning ability such as the exemplary level and comparison to a traditional teaching approach. Moreover, this study was designed based on the Clinical Judgment Model proposed by Tanner [4] with a specific focus on clinical reasoning ability. Future studies must consider various categories of outcome variables, such as nursing performance, and team collaboration.

## Conclusions

This study demonstrated that S-PBL strengthened nursing students’ clinical reasoning ability by repeatedly applying it and measuring its education effect and students’ learning satisfaction level was shown to be high. This suggests that nursing education using S-PBL is a good educational strategy and an effective teaching method that enhances students’ learning satisfaction. Future studies must compare clinical reasoning ability more objectively by establishing a control group considering various learner variables. Furthermore, repeat studies must be conducted using different educational techniques, especially by comparison to traditional teaching method, and strategies for effective and standardized design and S-PBL operation.

### Electronic supplementary material

Below is the link to the electronic supplementary material.

Supplementary Material 1

Supplementary Material 2

## Data Availability

The data presented in this study are available on request from the corresponding author. The data are not publicly available due to restrictions (privacy or ethical).

## List of abbreviations

ANOVA: Analysis of variance
GPA: Grade point average
LCJR: Lasater clinical judgment rubric
Ob/Gyn: Obstetrics-gynecology
S-PBL: Simulation with problem-based learning
