# Supplementary material for: Effects of simulation with problem-based learning (S-PBL) on nursing students’ clinical reasoning ability: based on Tanner’s clinical judgment model
Source: BMC Med Educ. 2023 Aug 24;23:601. doi: 10.1186/s12909-023-04567-9 (PMC10464450; doi:10.1186/s12909-023-04567-9)
Supplement: Supplementary file 2 — Supplementary Material 2 [file 12909_2023_4567_MOESM2_ESM.pdf]

JBI CRITICAL APPRAISAL CHECKLIST FOR QUASI-EXPERIMENTAL STUDIES

Reviewer___Hae Kyoung Son________________Date_____02 August, 2023_____

Author___Hae Kyoung Son___________________Year_2023___ Record Number_2022-061_

	Yes	No	Unclear	Not applicable
1. Is it clear in the study what is the 'cause' and what is the 'effect' (i.e. there is no confusion about which variable comes first)?	■	□	□	□
2. Were the participants included in any comparisons similar?	□	□	□	■
3. Were the participants included in any comparisons receiving similar treatment/care, other than the exposure or intervention of interest?	□	□	□	■
4. Was there a control group?	□	■	□	□
5. Were there multiple measurements of the outcome both pre and post the intervention/exposure?	■	□	□	□
6. Was follow up complete and if not, were differences between groups in terms of their follow up adequately described and analyzed?	■	□	□	□
7. Were the outcomes of participants included in any comparisons measured in the same way?	□	□	□	■
8. Were outcomes measured in a reliable way?	■	□	□	□
9. Was appropriate statistical analysis used?	■	□	□	□

Overall appraisal: Include ■ Exclude □ Seek further info □

Comments (Including reason for exclusion)

____

____

____
